# Tetrahydrocurcumin Ameliorates Cerebral Ischemia–Reperfusion Injury and Restores Blood–Brain Barrier Dysfunction by Inhibiting Ferroptosis

**DOI:** 10.1111/cns.70662

**Published:** 2025-11-21

**Authors:** Shuang Zhang, Jizhong Han, Zhen Fan, Haoxiang Wang, Luotong Liu, Liang Liu, Liangxue Zhou, Huajiang Deng

**Affiliations:** ^1^ Department of Geriatrics Affiliated Hospital of Southwest Medical University Luzhou Sichuan Province China; ^2^ Department of Neurosurgery Affiliated Hospital of Southwest Medical University Luzhou Sichuan Province China; ^3^ Department of Geriatrics, Sichuan Provincial People's Hospital University of Electronic Science and Technology of China Chengdu Sichuan Province China; ^4^ Department of Neurosurgery West China Hospital of Sichuan University Chengdu Sichuan Province China

**Keywords:** blood–brain barrier, cerebral ischemia–reperfusion injury, ferroptosis, neurological function, Nrf2, tetrahydrocurcumin

## Abstract

**Background:**

Cerebral ischemia–reperfusion (I/R) injury is a major consequence of ischemic stroke, leading to blood–brain barrier (BBB) disruption, neuroinflammation, and neuronal death. Recent studies suggest that tetrahydrocurcumin (THC), a natural compound, may have neuroprotective effects in ischemic stroke. However, the underlying mechanisms remain unclear. This study aims to investigate THC's neuroprotective effects in cerebral I/R injury and explore its potential mechanisms.

**Methods:**

A middle cerebral artery occlusion (MCAO) model was used to induce ischemia–reperfusion injury in mice. Bioinformatics analysis identified key genes involved in ferroptosis. THC's effects were assessed by evaluating infarct volume, BBB permeability, and ferroptosis‐related markers (GPX4, xCT, FTH1). Molecular mechanisms were explored using an Nrf2‐specific inhibitor (ML385) and molecular docking analysis.

**Results:**

THC treatment significantly reduced infarct volume, alleviated BBB disruption, and improved neurological function. It inhibited ferroptosis by upregulating the expression of GPX4, xCT, and FTH1, and by decreasing lipid peroxidation and iron accumulation. THC promoted Nrf2 nuclear translocation, which in turn activated the downstream antioxidant pathway. Molecular docking analysis revealed that THC binds to Keap1, promoting Nrf2 dissociation and nuclear translocation. ML385 reversed THC's protective effects, confirming the involvement of the Keap1/Nrf2 signaling pathway.

**Conclusion:**

THC inhibits ferroptosis through the activation of the Keap1/Nrf2 signaling pathway, significantly improving BBB dysfunction and alleviating neurological deficits following cerebral ischemia–reperfusion. These findings suggest that THC could serve as a potential therapeutic agent for ischemic stroke, providing a novel approach for the treatment of cerebral ischemia–reperfusion injury through ferroptosis modulation.

## Graphical abstract

Tetrahydrocurcumin (THC) inhibits ferroptosis and restores blood–brain barrier (BBB) function by activating the Keap1/Nrf2 signaling pathway, thereby alleviating neurological dysfunction induced by cerebral ischemia–reperfusion.

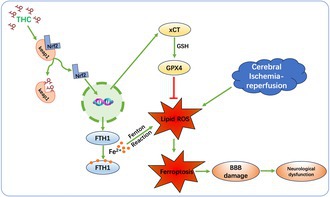

## 1 Introduction

Cerebral infarction (ischemic stroke) is one of the leading causes of death and disability worldwide, accounting for approximately 80% of all strokes [1]. It is the most prevalent type of stroke, particularly in societies with aging populations, where its incidence continues to rise, placing a substantial burden on public health and the economy. Although modern medicine provides treatment options such as intravenous thrombolysis (tPA) [2], and mechanical thrombectomy, these therapies have strict time window limitations and carry a high risk of hemorrhage. Furthermore, reperfusion can lead to additional injury, including oxidative stress and inflammatory responses, which result in antioxidant system failure, lipid peroxidation, and DNA damage. Due to their high metabolic and oxygen consumption rates and low antioxidant enzyme activity, neurons are particularly susceptible to lipid peroxidation‐induced damage compared to other cell types.

Ferroptosis is a novel type of cell death discovered in recent years, typically accompanied by excessive iron accumulation and lipid peroxidation [3]. Ferroptosis inducers can disrupt glutathione (GSH) synthesis, impair glutathione peroxidase 4 (GPX4) activity, and lead to the accumulation of lipid reactive oxygen species (ROS), resulting in oxidative stress and antioxidant system imbalance, ultimately causing cell death. Morphologically, ferroptosis is characterized by the loss of plasma membrane integrity, mitochondrial swelling, cristae reduction, and outer membrane rupture [4]. This pathway has been shown to play a critical role in the pathogenesis of various neurological diseases [5, 6, 7], suggesting that ferroptosis could serve as a novel therapeutic target in the treatment of cerebral infarction.

Tetrahydrocurcumin (THC) is one of the main metabolites of curcumin, characterized by its strong water solubility and high bioavailability [8]. It has attracted widespread attention due to its potent antioxidant properties. THC exerts neuroprotective effects primarily by scavenging free radicals, inhibiting lipid peroxidation, and enhancing the endogenous antioxidant system, such as upregulating superoxide dismutase (SOD) and GSH levels. It has been shown to alleviate oxidative stress and inflammation‐mediated damage in various neurological diseases [9]. In recent years, the potential role of THC in ischemic brain injury has gradually attracted attention. Studies have shown that THC can reduce brain edema, improve cerebral blood flow, and repair the blood–brain barrier (BBB) [10]. Studies have demonstrated that ferroptosis serves as a key driver of BBB disruption following stroke [11], and downregulation of the GPX4/xCT pathway has been shown to exacerbate endothelial cell injury [12]. Although the antioxidant properties of THC are closely related to the core pathological feature of ferroptosis—lipid peroxidation—whether THC exerts neuroprotective effects by modulating ferroptosis pathways remains to be clarified. As a crucial mechanism in ischemia–reperfusion injury, ferroptosis exhibits potential connections between the expression changes of its core regulatory molecules (GPX4 and xCT) and the antioxidant targets of THC. However, this mechanistic relationship has not yet been experimentally validated. To date, most studies on the neuroprotective effects of THC have focused on its antioxidative mechanisms [13, 14, 15], while its potential role in regulating ferroptosis has not been systematically investigated. This knowledge gap limits our comprehensive understanding of how THC exerts its protective effects in cerebral ischemic injury.

Therefore, this study aims to investigate the protective role of THC in cerebral I/R injury, with particular emphasis on its potential to inhibit ferroptosis and restore BBB function. The findings may help fill the existing gap in understanding the mechanistic link between THC and ferroptosis and provide novel experimental evidence supporting its therapeutic potential in ischemic stroke.

## 2 Materials & Methods

### 2.1 Animals

SPF‐grade, healthy adult male C57BL/6 mice (age 6–8 weeks, weight 17–25 g) were used in this study, provided by Hunan Silaikejingda Experimental Animal Co. Ltd. (license number SCXK (Xiang)‐2019–0004). The mice were housed in an SPF‐grade facility with controlled conditions: humidity at 38%–72%, room temperature at 22°C–26°C, and a 12 h light/dark cycle. Food and water were provided ad libitum. All experimental protocols were approved by the Animal Ethics Committee of Southwest Medical University (Approval No. 20241024–002). Animal experiments were conducted adhering to the ARRIVE guidelines. Mice were anesthetized with pentobarbital sodium (50 mg/kg, intraperitoneal injection). At the end of the experiment, mice were euthanized by intraperitoneal injection of an overdose of pentobarbital sodium (150 mg/kg) following the guidelines provided by the Animal Ethics Committee.

### 2.2 Middle Cerebral Artery Occlusion (MCAO) Model and Grouping

The middle cerebral artery ischemia–reperfusion model was established in mice as previously described [16]. Mice were anesthetized, and body temperature was maintained at 36.5°C–37.5°C. In the supine position, a midline incision was made in the anterior neck to bluntly separate the subcutaneous tissue, exposing the right common carotid artery, internal carotid artery (ICA), and external carotid artery (ECA). A small incision was made in the ECA, through which a 3 cm filament (φ = 0.126 mm, silicone‐coated tip φ = 0.25 ± 0.02 mm) was inserted into the ECA and advanced slowly into the right middle cerebral artery. The filament was removed after 60 min.

The mice were randomly assigned to the following groups: sham, MCAO, L‐THC (MCAO mice with 10 mg/kg THC treatment) group, H‐THC (MCAO mice with 25 mg/kg THC treatment) group, and THC+ ML385 (MCAO mice with 25 mg/kg THC and 30 mg/kg ML385 treatment) group. THC (Sigma‐Aldrich, SMB00370) and ML385 (Sigma‐Aldrich, SML1833) were given for 3 days by intraperitoneal injection after ischemia [10].

### 2.3 3, 5‐Triphenyl‐2H‐Tetrazolium Chloride (TTC) Staining

Three days after MCAO, the mice were euthanized, and the brain tissue was sectioned into five slices (approximately 1 mm thick). The sections were then incubated in 2% TTC solution at 37°C for 20 min. Following incubation, the slices were fixed in 4% paraformaldehyde. After successful TTC staining, viable tissue appeared red, while the infarcted areas appeared white. Infarct volume was quantified using ImageJ software.

### 2.4 BBB Permeability Assessment

A 2% Evans blue (EB) solution (4 mL/kg) was injected via the right femoral vein, and brains were collected 1 h later after saline perfusion. The lesioned and contralateral hemispheres were weighed and homogenized in formamide solution (100 mg/mL). The homogenates were incubated in a 60°C water bath for 24 h, then centrifuged at 5000 r/min for 20 min, followed by a second centrifugation at 10,000 r/min for 10 min after removing the supernatant. Finally, 200 μL of the supernatant was transferred to a microplate, and absorbance was measured at 630 nm.

In another set of mice, EB fluorescence intensity was measured using confocal laser scanning microscopy. Mice were euthanized 1 h after EB injection via the right femoral vein, and 20‐μm‐thick frozen brain sections were prepared. Sections were baked in a 50°C oven for 1 h and subsequently stained with DAPI.

### 2.5 Brain Water Content

After euthanizing the animals, the brain was quickly removed, and the meninges and surface blood were carefully cleared. Both cerebral hemispheres and the cerebellum were rapidly weighed using an electronic balance (accuracy of 0.1 mg) to obtain the wet weight. The samples were then placed in a 100°C oven for 24 h to determine the dry weight. Brain water content (%) was calculated using the formula: Brain water content (%) = (wet weight‐dry weight)/wet weight × 100.

### 2.6 Bioinformatics Analysis

The RNA‐seq dataset GSE163752 was downloaded from the GEO database (https://www.ncbi.nlm.nih.gov/geo/). This dataset contains endothelial cells isolated from the contralateral hemisphere and the ipsilateral hemisphere (1 h occlusion followed by 23 h of reperfusion) of mouse brains. Each sample includes six biological replicates, with each replicate consisting of 3–4 male C57BL/6 mice. Differentially expressed genes (DEGs) between the two groups were identified using the limma package. DEGs were defined as genes with a *p* < 0.05 and an absolute |log2FC| > 1. Gene Ontology (GO) and Kyoto Encyclopedia of Genes and Genomes (KEGG) pathway enrichment analyses for the DEGs were performed using the clusterProfiler package. Results with an adjusted *p* < 0.05 were considered significantly enriched. For candidate genes, Protein–Protein Interaction (PPI) analysis was conducted based on the STRING database (https://STRING‐db.org/). Network construction was performed using Cytoscape software, and key genes were screened using the cytoHubba and MCODE algorithms. The venn package was used to identify the intersection of key genes obtained by both algorithms.

### 2.7 Docking Structure of Target Protein and Small‐Molecule Drug

The three‐dimensional structure of the target protein was downloaded from the PDB database (https://www.rcsb.org/), and the structure of the small‐molecule drug was obtained from the PubChem database (https://pubchem.ncbi.nlm.nih.gov/). Molecular docking of the selected ligand with the target protein was conducted using CB‐Dock2 (https://cadd.labshare.cn/cb‐dock2/php). The complex model with the highest docking score was visualized and analyzed using PymolWin software.

### 2.8 Neurological Function Score

To ensure the reliability of the scoring results, three reviewers were employed for blinded evaluation, and the average score for each animal was calculated. Neurological function was assessed using the Garcia scoring system, which includes the following criteria: (1) spontaneous activity, (2) symmetry of movement, (3) symmetry of forelimb movement, (4) ability to climb the wire mesh cage wall, (5) response to tactile stimulation on both sides of the trunk, and (6) response to whisker touch. Lower scores indicate more severe neurological impairment.

### 2.9 Morris Water Maze

During the training phase, mice were trained to locate a hidden platform within 60 s and remain on it for 10 s. If the platform was not found within the allotted time, the mice were guided to the platform and stayed for 10 s. Each day, the mice were trained four times, once from each quadrant (first to fourth). In the testing phase, the platform was removed, and data were recorded on each mouse's movement trajectory, number of platform crossings, and time spent in each quadrant within the specified time.

### 2.10 Co‐Immunoprecipitation

Immunoprecipitation was performed using the Pierce Classic IP Kit, following the manufacturer's instructions. Briefly, cells were lysed in ice‐cold IP lysis buffer with protease inhibitors. Samples were incubated overnight at 4°C with a specific antibody to form immune complexes, which were then combined with Protein A/G Plus agarose to remove unbound substances. The bound immune complexes were separated from Protein A/G using a low‐pH elution buffer and analyzed by Western blotting.

### 2.11 Prussian Blue Staining

After deparaffinization and gradual rehydration, paraffin sections were rinsed with water for 5 min and subjected to antigen retrieval. Staining was then performed following the instructions for the Prussian Blue Staining Kit (Servicebio, G1029). The sections were then gradually dehydrated and finally mounted with neutral resin.

### 2.12 Transmission Electron Microscopy (TEM)

The sample was fixed, dehydrated, and embedded. Ultra‐thin sections of approximately 90 nm were prepared, floated onto grids, and stained with uranyl acetate for 10 min and lead citrate for 2 min at room temperature. Images were acquired with a transmission electron microscope to observe the relevant pathological changes.

### 2.13 Measurement of GSH, SOD, Malondialdehyde (MDA), and Iron

The brain tissue was homogenized in PBS, and the supernatant was collected after high‐speed centrifugation for subsequent measurements. The levels of MDA (Servicebio, G4302), SOD (Servicebio, G4306), GSH (Servicebio, G4305), and iron (Abcam, ab83366) in the brain tissue were measured according to the instructions provided in the reagent kit.

### 2.14 Immunofluorescence

After perfusion and brain extraction, paraffin sections were prepared from fixed and dehydrated brain tissue for immunofluorescence staining. The paraffin sections were deparaffinized, rehydrated, and subjected to antigen retrieval. The sections were blocked with 10% normal goat serum at room temperature for 1 h, then incubated overnight at 4°C with primary antibodies: anti‐CD31 (mouse, 1:600, Servicebio, GB12063‐100), anti‐GPX4 (rabbit, 1:250, Servicebio, GB115275‐50). Subsequently, secondary antibodies were incubated at room temperature for 2 h, followed by DAPI staining for 10 min. Observations were made under an inverted fluorescence microscope (BX53, Olympus).

### 2.15 Separation and Preparation of Nuclear and Cytoplasmic Proteins

According to the instructions of the Nuclear and Cytoplasmic Protein Extraction Kit (P0028 Beyotime), tissue samples were homogenized in ice‐cold conditions and mixed with cytoplasmic protein extraction reagents A/B (20:1 ratio) containing PMSF. After incubation on ice for 15 min, the homogenate was centrifuged at 1500 g for 5 min at 4°C to collect the supernatant. The pellet was resuspended in 200 μL of reagent A, vortexed vigorously for 5 s, and incubated on ice for 10–15 min. Then, 10 μL of reagent B was added, followed by another 5 s vortex and 1 min ice incubation. The mixture was centrifuged at 12,000–16,000 g for 5 min at 4°C. The supernatant was collected as the cytoplasmic protein. The remaining pellet was lysed with 50 μL of nuclear extraction reagent with intermittent vortexing for 30 min, followed by centrifugation at 12,000–16,000 g for 10 min at 4°C to collect the nuclear protein fraction. All procedures were performed on ice, with care taken to avoid disturbing the pellet during supernatant transfer.

### 2.16 Western Blot (WB) Analysis

After euthanizing the mice, brain tissue was quickly harvested, and total protein was extracted from the infarct region. Proteins were transferred onto polyvinylidene fluoride (PVDF) membranes (Millipore, Billerica, MA, USA) and blocked with 5% bovine serum albumin (BSA; Servicebio) at room temperature for 1 h. The membranes were incubated overnight at 4°C with primary antibodies: anti‐xCT (rabbit, 1:1000, Thermo Fisher, 711,589), anti‐GPX4 (rabbit, 1:1000, Servicebio, GB115275‐50), anti‐occludin (rabbit, 1:1000, Servicebio, GB111401), anti‐ACSL4 (rabbit, 1:1000, Abcam, ab155282), anti‐FTH1 (rabbit, 1:1000, Abcam, ab183781), anti‐Nrf2 (rabbit, 1:1000, Proteintech, 80,593–1‐RR), and anti‐Keap1 (mouse, 1:1000, Abcam, ab119403). The internal reference proteins included anti‐Tubulin (rat, 1:1000, Abcam, ab6161), anti‐GAPDH (mouse, 1:1000, Servicebio, GB15002), and anti‐TBP (mouse, 1:1000, Abcam, ab300656). The membranes were then incubated with secondary antibodies: HRP‐conjugated Affinipure Goat Anti‐Mouse IgG (H + L) (1:8000, Proteintech, SA00001‐1), HRP‐conjugated Affinipure Goat Anti‐Rabbit IgG (H + L) (1:8000, Proteintech, SA00001‐2), and HRP‐conjugated Affinipure Goat Anti‐Rat IgG (H + L) (1:8000, Proteintech, SA00001‐15). Enhanced chemiluminescence (Millipore Sigma) was used to visualize the protein bands, and ImageJ software was used for grayscale analysis of the bands.

### 2.17 Statistical Analysis

Statistical and image analyses of all experimental data were conducted using GraphPad Prism 9.0 and ImageJ software. All data were presented as mean ± standard deviation. The normality of data distribution was assessed using the Shapiro–Wilk test. Normally distributed continuous variables were compared between two groups with Student's t‐test, while non‐normally distributed variables were analyzed using the Mann–Whitney test. For multi‐group comparisons involving three or more groups, normally distributed data were evaluated through one‐way analysis of variance (ANOVA) with Bonferroni post hoc testing, whereas non‐normally distributed data were examined using the Kruskal‐Wallis test. *p* < 0.05 was considered statistically significant. All experiments were independently repeated at least three times (*n* ≥ 3) to confirm reproducibility.

## 3 Result

### 3.1 THC Improves BBB Dysfunction and Alleviates Brain Injury After MCAO

**FIGURE 1:**
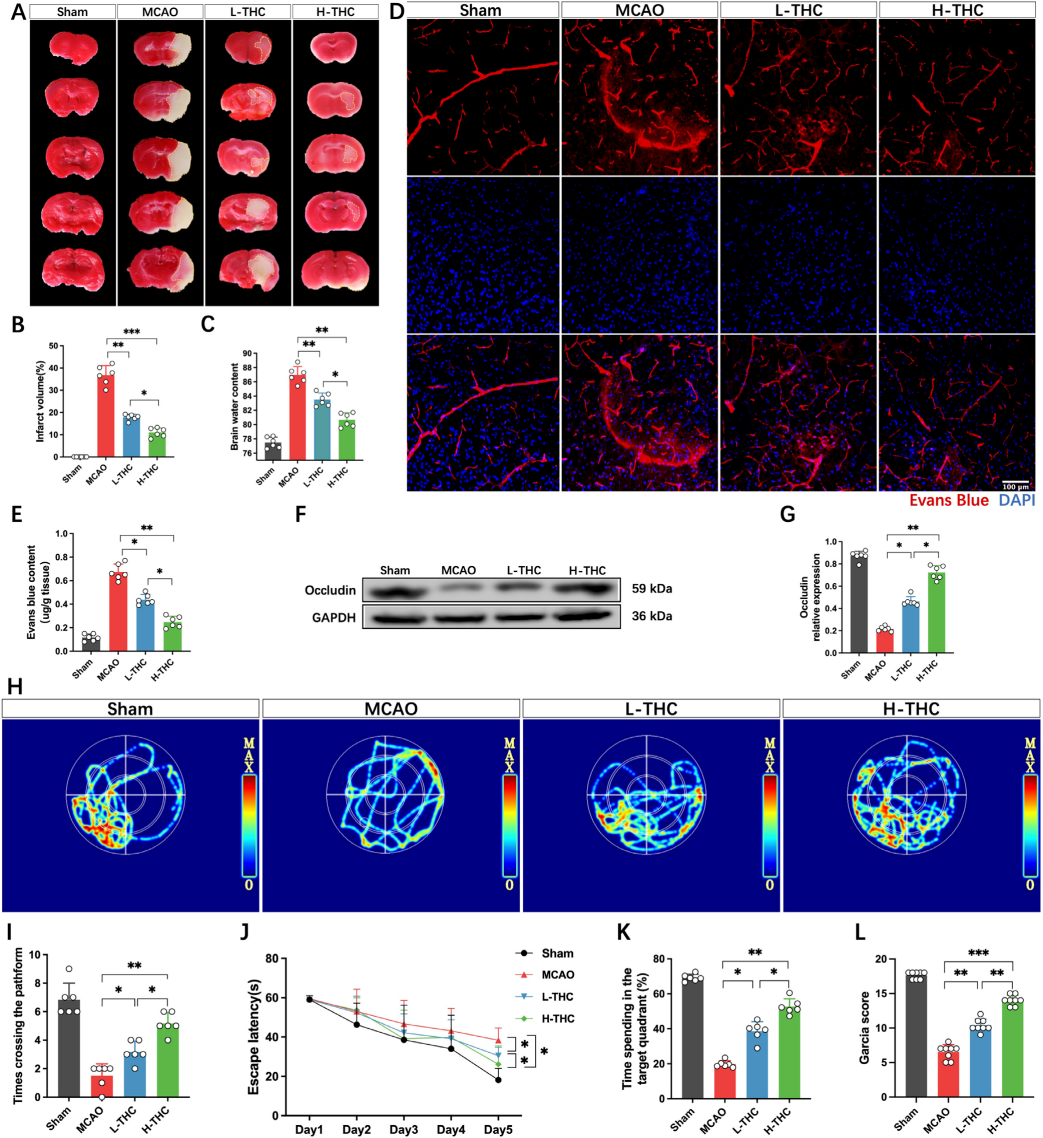
THC improves BBB integrity and alleviates brain injury after MCAO. (A) Representative TTC‐stained coronal brain sections, with red indicating normal brain tissue and white indicating infarcted tissue. The yellow‐dashed line marks the infarct area. (B) Analysis of infarct volume. (C) Quantitative analysis of brain water content in each group. (D) Laser confocal imaging analysis of EB leakage. (E) Analysis of EB content in brain tissue. (F and G) Western blot images and quantitative analysis of occludin protein levels. (H) Heatmap of swimming trajectories in the Morris Water Maze test. (I–K) Data analysis charts from the Morris Water Maze test. (L) Neurological function scores. All data are presented as mean ± SD, *n* = 6–8 per group, one‐way ANOVA, *p < 0.05, **p < 0.01, ***p < 0.001.

The results showed consistent trends across experimental batches. THC exhibited significant biological activity and low toxicity [8]. In this experiment, the therapeutic dose was based on previous studies [10, 17, 18], and no drug‐related abnormal behavioral changes, seizures, or deaths were observed during the experiment. TTC staining results showed that mice subjected to MCAO displayed a large infarct area and significant brain edema, while THC‐treated mice showed a significant reduction in infarct volume and marked alleviation of brain edema. The efficacy of the H‐THC group was superior to that of the L‐THC group (Figure 1A–C). Considering the critical role of the BBB in brain injury, its integrity was assessed using EB dye. The results indicated that all experimental groups exhibited varying degrees of EB leakage following MCAO. However, compared to the MCAO group, both L‐THC and H‐THC groups showed significantly reduced EB leakage, with the H‐THC group demonstrating a greater reduction than the L‐THC group (Figure 1D,E). Since the degradation of tight junction proteins is a key factor in BBB damage after MCAO, the expression of occludin was further assessed. The results showed that occludin protein levels were significantly downregulated in the brains of MCAO mice, while THC treatment restored occludin expression, with the H‐THC group showing better efficacy than the L‐THC group (Figure 1F,G). Using the Garcia scoring system to assess neurological function, significant neurological impairment was observed in mice after cerebral ischemia–reperfusion, including contralateral limb paralysis and reduced mobility. Additionally, the water maze test showed severe impairment in spatial exploration and memory abilities. However, THC treatment significantly improved neurological function in mice with ischemia–reperfusion injury, with the H‐THC group showing significantly better results than the L‐THC group (Figure 1H–L).

### 3.2 The Ferroptosis Pathway Mediates Endothelial Cell Injury in Cerebral Vessels After MCAO

**FIGURE 2:**
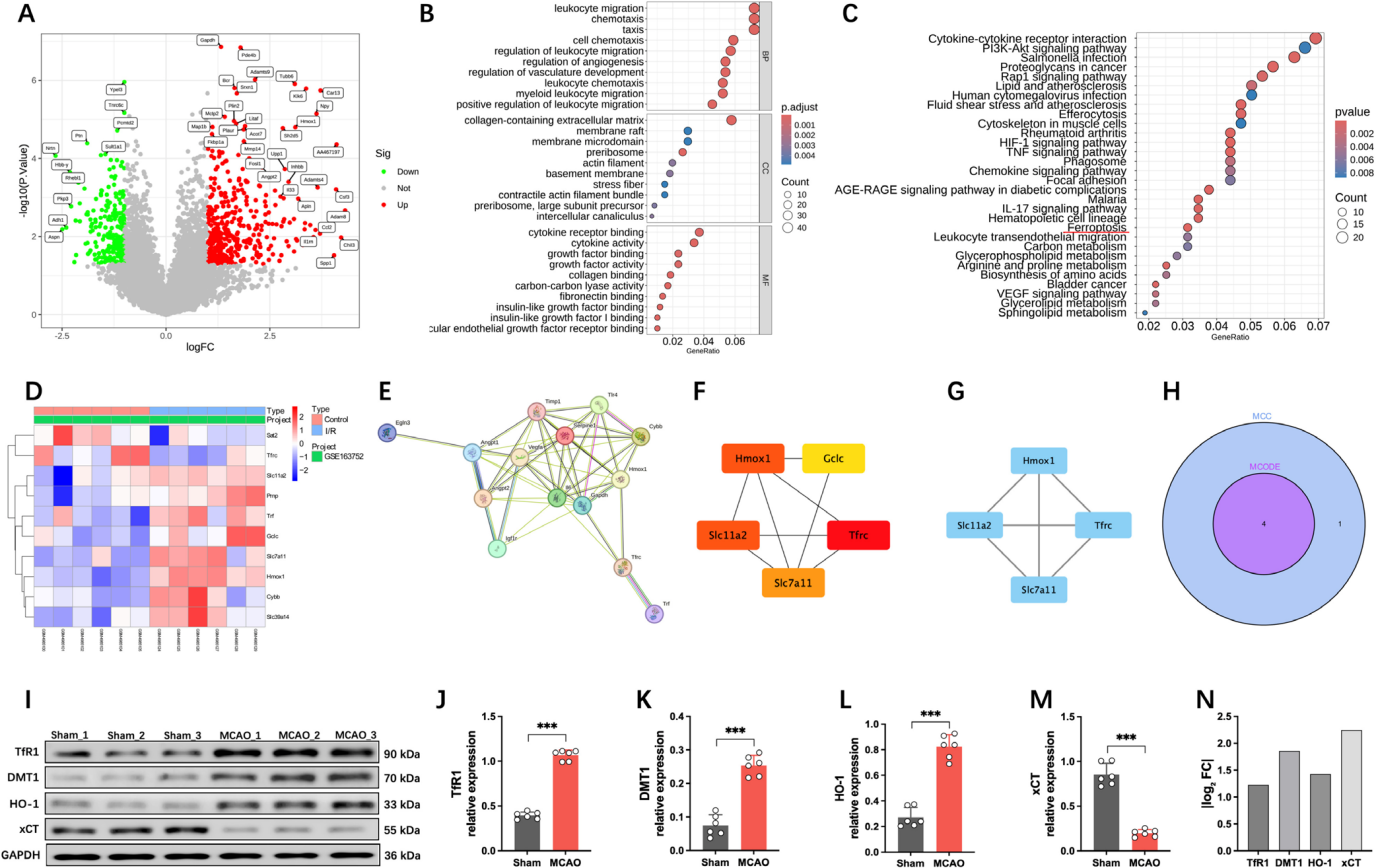
Bioinformatics analysis. (A) Volcanic map of differentially expressed genes. (B) GO enrichment analysis of differentially expressed genes. (C) KEGG enrichment analysis of differentially expressed genes. (D) Heat map of gene expression related to the ferroptosis pathway. (E) PPI interaction network of genes related to the ferroptosis pathway. (F) MCC algorithm TOP5 key gene networks. (G) MCODE algorithm is used to screen key gene networks. (H) Intersection of two algorithm results. (I‐M) Western blot images and quantitative analysis of TfR1, DMT1, HO‐1, and xCT protein expression. (N) Quantification of protein expression levels of TfR1, DMT1, HO‐1, and xCT based on |log2 Fold Change|. All data are presented as mean ± SD, *n* = 6 per group, unpaired Student's t‐test, ***p < 0.001.

Cerebral vascular endothelial cells, as a crucial component of the BBB, play a vital role in maintaining its structural and functional integrity. Therefore, we conducted bioinformatics analysis on endothelial cells within the infarcted brain tissue of mice. A total of 632 DEGs were identified from the GSE163752 dataset, with 396 upregulated and 236 downregulated (Figure 2A). GO enrichment analysis revealed that these DEGs are primarily involved in immune responses, extracellular matrix organization, and growth factor regulation (Figure 2B), with significant enrichment in pathways related to cell adhesion, hypoxic stress, and ferroptosis (Figure 2C,D). Based on the STRING database (https://STRING‐db.org/), we performed a PPI analysis of 10 key genes associated with the ferroptosis pathway, resulting in 10 protein nodes and 15 interactions (Figure 2E). Subsequently, we used the cytoHubba and MCODE algorithms to screen for key genes (Figure 2F,G), obtaining an intersection of the two methods and identifying four critical genes: HMOX1, SLC7A11, SLC11A2, and TFRC (Figure 2H). Among these four genes, SLC7A11 (xCT) plays a central role in ferroptosis by directly regulating System Xc^−^‐mediated cystine uptake, maintaining GSH synthesis and GPX4 activity, thereby inhibiting lipid peroxidation. While TFRC (TfR1) and SLC11A2 (DMT1) are involved in iron metabolism, they act as upstream indirect regulators of ferroptosis. HMOX1 (HO‐1) exhibits dual roles (promoting/anti‐ferroptosis) and lacks specificity [19]. Moreover, Western blot analysis revealed that xCT showed the most significant expression change in the MCAO mouse model (Figure 2I–N). Therefore, SLC7A11 was selected as the primary research target due to its direct and essential role in ferroptosis.

### 3.3 THC Mitigates Ferroptosis Following After MCAO

**FIGURE 3:**
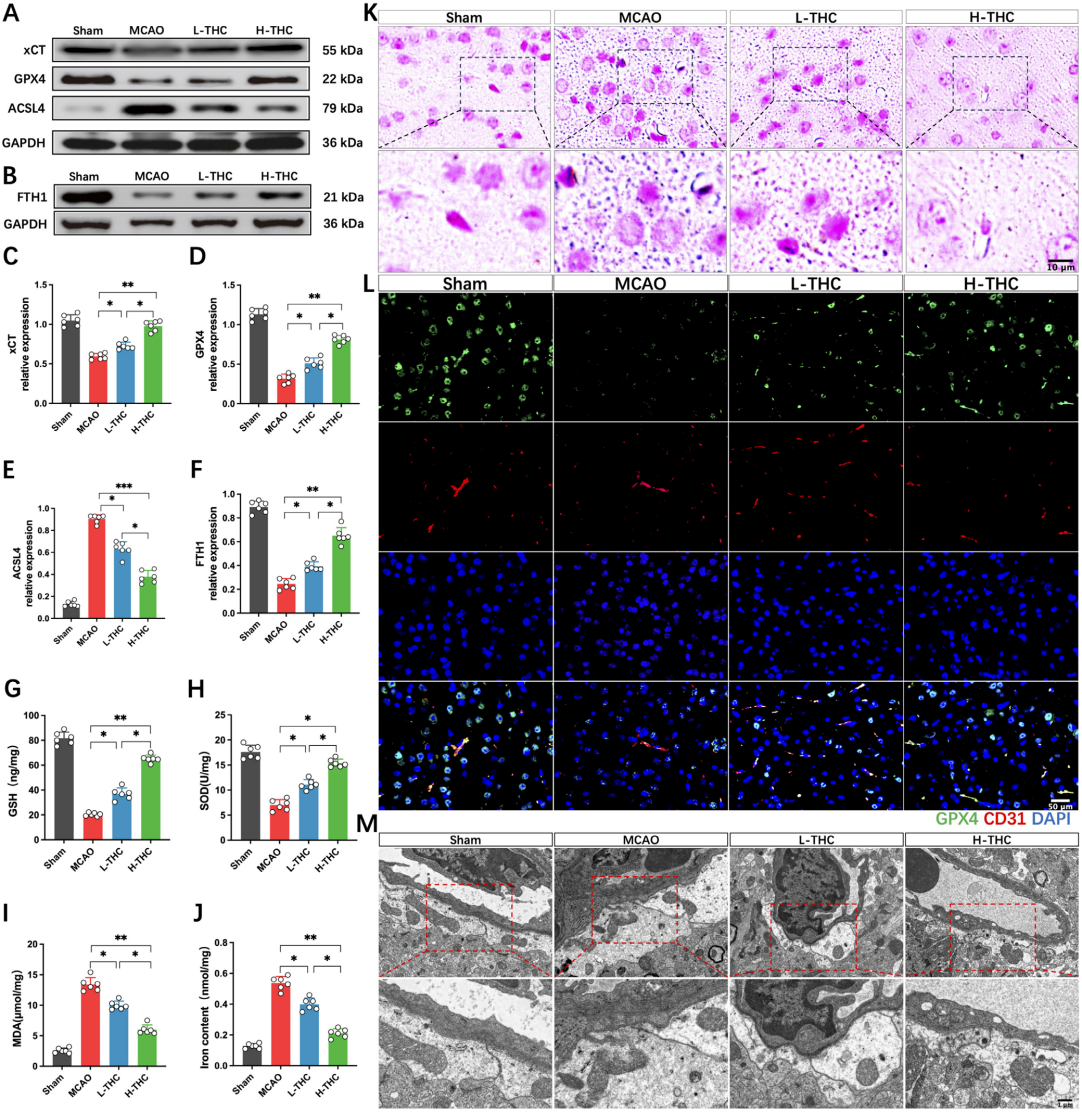
THC mitigates ferroptosis after MCAO. (A–F) Western blot images and quantitative analysis of xCT, GPX4, ACSL4, and FTH1 protein expression. (G–J) Quantitative analysis of iron content, GSH, SOD, and MDA levels in brain tissue. (K) Representative images of Prussian blue staining. (L) Co‐immunostaining of CD31 and GPX4 in cerebral microvessels. (M) Ultrastructural changes in the BBB. All data are presented as mean ± SD, *n* = 6 per group, one‐way ANOVA, *p < 0.05, **p < 0.01, ***p < 0.001.

Based on the previous bioinformatics analysis, we further validated the role of ferroptosis in cerebral infarction and explored the regulatory effect of THC on ferroptosis by examining the changes in ferroptosis‐related biomarkers using Western blot. The results showed that MCAO led to a significant downregulation of xCT, GPX4, and FTH1 proteins, while the expression of ACSL4, a key protein involved in lipid peroxidation, was significantly increased. In the L‐THC and H‐THC groups, compared to the MCAO group, the levels of xCT, GPX4, and FTH1 were significantly upregulated, and ACSL4 expression was decreased, with the H‐THC group showing a more significant effect than the L‐THC group (Figure 3A–F). Additionally, MCAO led to an increase in MDA levels, a decrease in GSH and SOD levels, and a significant increase in iron content and deposition in brain tissue (Figure 3G–J). Prussian blue staining further revealed that MCAO induced extensive iron deposition in neurons and endothelial cells, which was significantly reversed by THC treatment, with the H‐THC group showing a superior effect compared to the L‐THC group (Figure 3K). Immunofluorescence staining of brain vasculature showed that MCAO significantly decreased the expression of GPX4, a ferroptosis marker, in endothelial cells, while THC treatment alleviated this process, with the H‐THC group showing better results (Figure 3L). TEM further revealed ultrastructural changes in the BBB, showing that MCAO induced shrinkage of mitochondria, increased membrane density, outer membrane rupture, and the loss of mitochondrial cristae in endothelial cells and astrocyte endfeet. THC treatment significantly alleviated mitochondrial damage, and the H‐THC group showed better results than the L‐THC group (Figure 3M). These results suggest that cerebral ischemia–reperfusion injury activates ferroptosis, and THC treatment effectively alleviates ferroptosis induced by MCAO.

### 3.4 THC Enhances Nrf2 Nuclear Translocation by Inhibiting Keap1‐Nrf2 Binding

**FIGURE 4:**
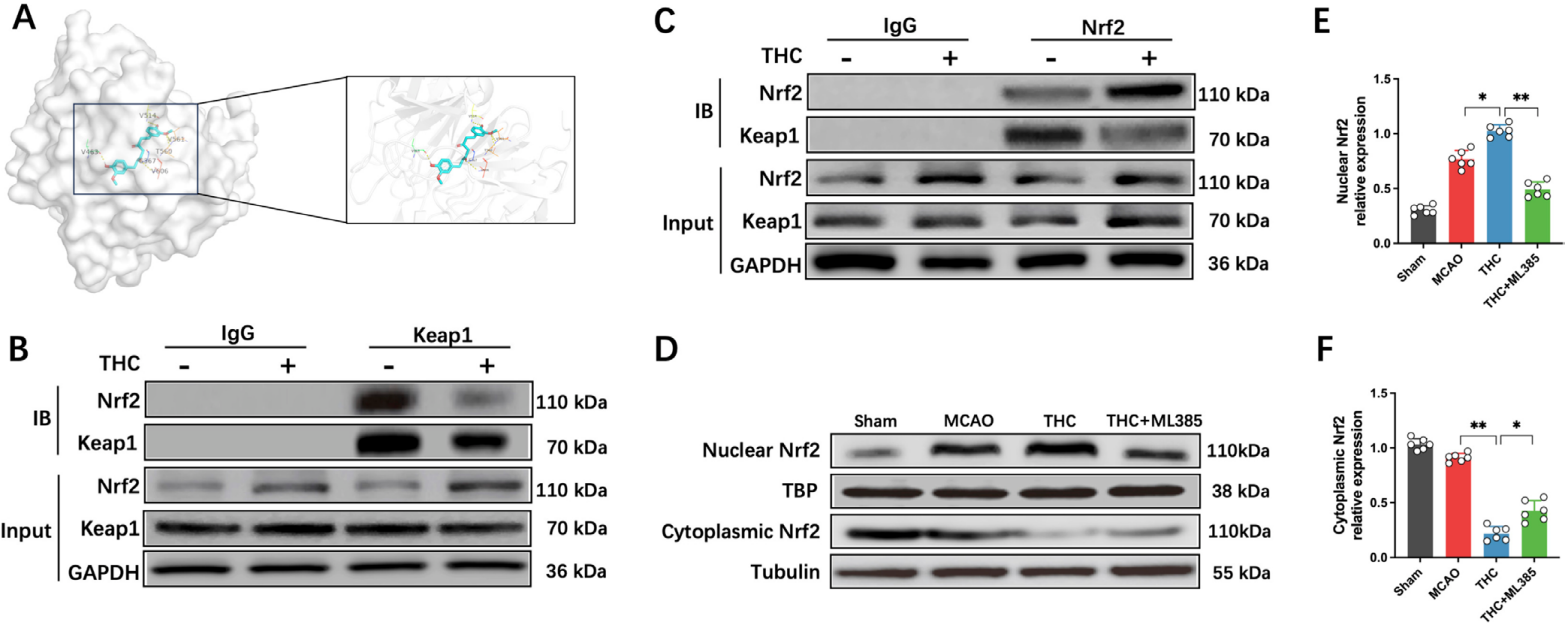
THC promoting Nrf2 nuclear translocation. (A) Molecular docking conformation of THC with Keap1. (B and C) Co‐immunoprecipitation analysis showing the interaction between Keap1 and Nrf2. (D–F) Western blot images and quantitative analysis of Nrf2 protein expression. All data are presented as mean ± SD, *n* = 6 per group, one‐way ANOVA, *p < 0.05, **p < 0.01, ***p < 0.001.

Nrf2 is a critical regulatory signaling pathway in GPX4‐dependent ferroptosis [20]. Previous studies have confirmed that under physiological conditions, Keap1 interacts with Nrf2 to form a complex, inhibiting Nrf2 nuclear translocation and promoting its ubiquitination and degradation. Under oxidative stress, Nrf2 dissociates from Keap1 and translocates to the nucleus to exert regulatory functions [21]. Proteins and small molecules can activate the Nrf2 pathway by competitively binding to Keap1 [22]. Therefore, we investigated whether THC interferes with the Keap1–Nrf2 interaction. Molecular docking analysis showed that THC binds to Keap1 at the Gly367, Val463, Val514, Thr560, Val561, and Val606 sites via hydrogen bonding, with the highest binding affinity of −8.2 kcal/mol, suggesting a strong binding affinity (Figure 4A). We further confirmed through co‐immunoprecipitation that THC reduced the interaction between Keap1 and Nrf2, promoting Nrf2 activation (Figure 4B,C). Western blot results showed that, compared to the MCAO group, the THC treatment group exhibited significantly increased nuclear Nrf2 expression, while cytoplasmic Nrf2 levels were markedly reduced (Figure 4D–F). This indicates that THC treatment significantly enhanced Nrf2 nuclear translocation.

### 3.5 THC Inhibits Ferroptosis and Restores BBB Function via the Nrf2 Signaling Pathway

**FIGURE 5:**
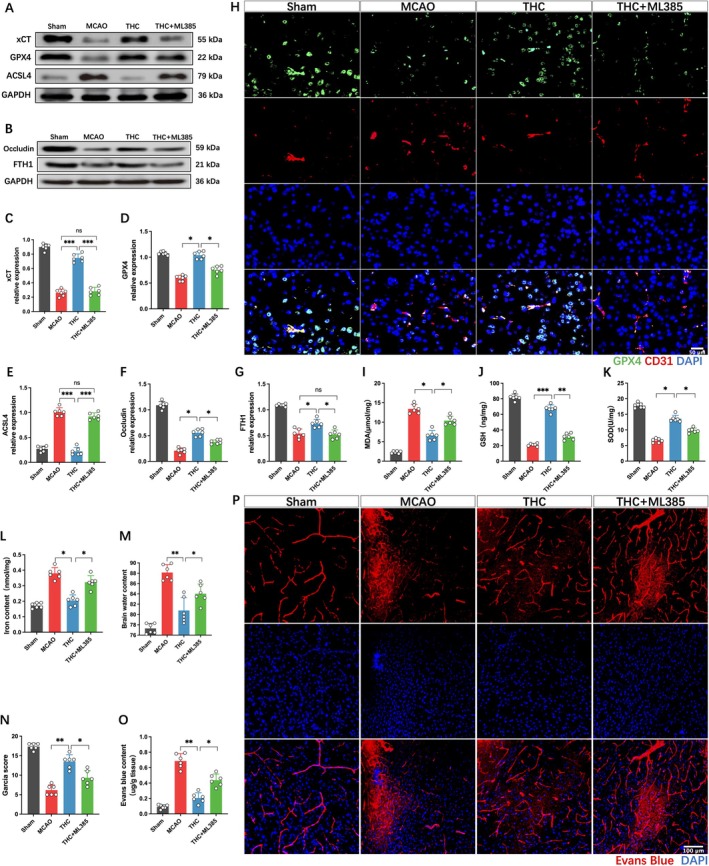
THC inhibits ferroptosis and restores BBB function via the Nrf2 signaling pathway. (A–G) Western blot images and quantitative analysis of xCT, GPX4, ACSL4, FTH1, and occludin protein expression. (H) Immunofluorescence staining of CD31 and GPX4 in brain microvessels. (I–L) Quantitative analysis of GSH, SOD, MDA levels, and iron content in brain tissue. (M) Quantitative analysis of brain water content in each group. (N) Neurological function scores. (O) Analysis of EB content in brain tissue. (P) Laser confocal imaging analysis of EB leakage. All data are presented as mean ± SD, *n* = 6 per group, one‐way ANOVA, *p < 0.05, **p < 0.01, ***p < 0.001.

Western blot analysis revealed that THC significantly enhanced the expression of ferroptosis‐related proteins, such as xCT, GPX4, FTH1, and tight junction protein occludin, while downregulating the protein expression of ACSL4, compared to the MCAO group (Figure 5A–G). THC also increased the levels of GSH and SOD, and decreased MDA levels and iron content (Figure 5I–L). Conversely, the addition of ML385 significantly reversed these effects, manifested as the downregulation of xCT, GPX4, FTH1, occludin, GSH, and SOD, and the upregulation of ACSL4, iron, and MDA. Immunofluorescence staining further indicated that THC promoted GPX4 expression in brain endothelial cells, exhibiting antioxidant activity, which was inhibited by ML385 (Figure 5H). Subsequently, we further evaluated the neurological function scores, brain water content, and BBB permeability in each group of mice. The results showed that ML385 could significantly reverse the protective effects of THC, manifesting as more severe neurological dysfunction, markedly aggravated cerebral edema, and enhanced EB leakage (Figure 5M–P).

To further verify the specificity of THC's mechanism, we compared its effects with the ferroptosis inhibitor Fer‐1 and the apoptosis inhibitor Z‐VAD in the OGD/R model. THC showed similar protective effects to Fer‐1 and was more effective than Z‐VAD in preserving cell viability. THC also markedly upregulated ferroptosis‐related proteins GPX4 and xCT, with only a modest effect on cleaved caspase‐3 (Figure S1), suggesting its BBB protection mainly involves ferroptosis inhibition.

## 4 Discussion

Ischemic stroke remains one of the leading causes of mortality and disability worldwide, yet current treatment strategies remain limited. Although reperfusion is an effective approach to salvage ischemic brain tissue [23], it may paradoxically induce ischemia–reperfusion (I/R) injury, characterized by oxidative stress, neuroinflammation, and apoptosis, ultimately leading to BBB disruption and secondary brain damage, thereby contributing to poor outcomes [24, 25, 26]. In this study, THC treatment significantly restored occludin expression, reduced EB extravasation and cerebral edema, and improved neurological deficits in the MCAO mouse model. These findings suggest that THC may hold therapeutic potential in preserving BBB integrity and mitigating cerebral I/R injury.

Ferroptosis, a novel form of programmed cell death driven by lipid peroxidation and iron accumulation, has recently garnered significant attention in stroke pathophysiology [27]. Excessive iron can promote lipid peroxidation of cell membranes through the Fenton reaction, thereby exacerbating neuronal damage and death. Ferritin (e.g., FTH1) plays a crucial buffering role in maintaining low levels of free and redox‐active iron [28], and its downregulation may accelerate the ferroptotic process [29]. In this study, we observed increased Fe^2+^ levels in brain tissue following MCAO, along with decreased levels of antioxidants (GSH), free radical scavengers (SOD), and FTH1 expression, accompanied by mitochondrial structural damage, suggesting that ferroptosis may play an important role in I/R injury.

Endothelial cells, as essential components of the BBB, serve as key regulatory sites for cerebral iron uptake and are tightly regulated by astrocytes [30]. Cerebral microvascular endothelial cells are particularly sensitive to oxidative stress and ferroptosis, and their damage can directly compromise BBB integrity. In this study, bioinformatic analysis indicated that ferroptosis is involved in the pathological injury of cerebral microvascular endothelial cells induced by cerebral ischemia–reperfusion. Consistently, we observed evidence of ferroptosis in cerebral endothelial cells in both in vitro and in vivo ischemia/hypoxia models, further supporting its critical role in BBB disruption. Notably, THC intervention markedly reversed these pathological alterations, enhanced antioxidant capacity, improved mitochondrial structure, and reduced iron overload, suggesting that its protective effects may be closely associated with the inhibition of ferroptosis.

In addition, SLC7A11 was identified through transcriptomic analysis as a key regulatory gene involved in ferroptosis of cerebral microvascular endothelial cells, with its expression markedly downregulated following ischemia. The xCT protein encoded by SLC7A11 is an important component of the Xc^−^ transport system, responsible for the uptake of cysteine to maintain intracellular GSH levels. The GSH‐GPX4 system constitutes the main defense mechanism against lipid peroxidation. Depletion of GSH or inactivation of GPX4 can lead to uncontrolled accumulation of lipid peroxides, thereby triggering ferroptosis [31]. Meanwhile, ACSL4, a crucial enzyme in fatty acid metabolism, could promote the esterification of polyunsaturated fatty acids and provide substrates for lipid peroxidation, further exacerbating ferroptosis. Notably, THC treatment effectively reversed these pathological changes and inhibited the ferroptosis cascade, suggesting that its protective effect on BBB integrity may be closely related to the suppression of ferroptosis.

The Keap1‐Nrf2 system is a central mechanism regulating cellular antioxidant defense. Nrf2 is a key regulatory factor in cellular antioxidant responses. Most genes associated with ferroptosis are regulated by Nrf2, including those involved in glutathione synthesis (e.g., system Xc^−^ and GPX4) and iron metabolism (e.g., FTH1 and FTL) [32, 33, 34]. Previous studies have shown that activation of Nrf2 can alleviate neuroexcitotoxicity and inhibit ferroptosis by downregulating ACSL4 expression [35]. In the present study, we found that THC may specifically bind to several critical sites on Keap1, thereby effectively disrupting the Keap1‐Nrf2 interaction, promoting Nrf2 nuclear translocation, and enhancing the expression of downstream target genes (such as SLC7A11 and GPX4). This mechanism not only explains the ability of THC to reduce iron accumulation and lipid peroxidation in brain tissue but also aligns with its observed enhancement of antioxidant capacity, including increased GSH and SOD levels. Notably, the protective effects of THC were markedly reversed by the Nrf2 inhibitor ML385, further supporting the pivotal role of this pathway in THC's mechanism of action. Our study provides the first demonstration in cerebral microvascular endothelial cells that THC may preserve BBB function through ferroptosis inhibition.

This study has several limitations. Although THC may exert its effects by binding to Keap1, its precise molecular targets remain unclear, which could be further verified through gene knockout or plasmid transfection approaches. Moreover, despite its superior stability and antioxidant capacity compared to curcumin, the clinical translation of THC still faces challenges, including low oral bioavailability, variable pharmacokinetic profiles, and limited safety data. Preliminary toxicological studies have shown no significant toxicity at high doses in rodents, suggesting a favorable safety profile. However, systematic and long‐term toxicological evaluations are still required. Future research should focus on elucidating the pharmacokinetics of THC in humans, exploring advanced delivery strategies (e.g., liposomes, nanoparticles) to enhance its bioavailability, and advancing early‐phase clinical trials to assess its efficacy and safety. Additionally, combination therapy strategies may further potentiate its therapeutic effects and broaden its clinical applications.

In conclusion, this study explored the neuroprotective mechanisms of THC in cerebral ischemia–reperfusion injury. The findings reveal for the first time that THC inhibits ferroptosis through the activation of the Keap1/Nrf2 signaling pathway, significantly improving BBB dysfunction and alleviating neurological deficits following cerebral ischemia–reperfusion. These results not only support the potential application of THC as a therapeutic agent for ischemic stroke but also lay a foundation for its clinical translation and broader applications.

## Author Contributions

Shuang Zhang conducted the study design, optimized experiments, interpreted data, and wrote the manuscript. Zhen Fan and Liangxue Zhou performed data collection and manuscript writing. Luotong Liu and Liang Liu conducted statistical analysis and data interpretation. Jizhong Han and Haoxiang Wang collected and organized data. Huajiang Deng contributed to the study design and data interpretation, and reviewed and revised the manuscript. All authors contributed to the article, and all read and approved the final manuscript.

## Funding

This work was supported by the Key R&D Technology Plan Project of Luzhou City (2024SYF134), the Natural Science Project of Southwest Medical University (2024ZKY005), the National Natural Science Foundation of China (82301771), Scientific Research Project of Sichuan Cadre Health Committee (2024–207).

## Conflicts of Interest

The authors declare no conflicts of interest.

## Supporting information


**Data S1:** Supporting Information.

## Data Availability

The data that support the findings of this study are available from the corresponding author upon reasonable request.
