# Supplementary material for: Tetrahydrocurcumin Ameliorates Cerebral Ischemia–Reperfusion Injury and Restores Blood–Brain Barrier Dysfunction by Inhibiting Ferroptosis
Source: CNS Neurosci Ther. 2025 Nov 21;31(11):e70662. doi: 10.1111/cns.70662 (PMC12639186; doi:10.1111/cns.70662)
Supplement: Supplementary file 1 — Data S1: Supporting Information. [file CNS-31-e70662-s001.pdf]

Supplementary Materials

1. Supplementary Methods

Cell culture and OGD/R model

Mouse brain microvascular endothelial cells (bEnd.3, ZQ-0090, Shanghai Zhong Qiao Xin Zhou Biotechnology Co., China) were cultured in DMEM supplemented with 10% FBS and 1% penicillin-streptomycin. OGD/R was performed by replacing the medium with glucose-free DMEM, DMEM, and cells were incubated in a hypoxic chamber (1% O_2 , 5% CO_2 , 94% N_2) at 37 °C for 8 hours. f Reoxygenation was achieved by returning cells to normal DMEM and incubating under normoxic conditions for 24 hours.

Drug treatment

Cells were pretreated with tetrahydrocurcumin (THC, 15 μM; HY-N0893, MedChemExpress, USA), ferrostatin-1 (Fer-1, 20 μM; T6500, TargetMol, China), or the apoptosis inhibitor Z-VAD-FMK (Z-VAD, 100 μM; S7023, Selleck, USA) 2 hours prior to OGD induction. Treatments were maintained throughout the reoxygenation phase. A vehicle control group was included.

Cell viability assay

Cell viability was assessed using the Cell Counting Kit-8 (CCK-8; G4103-5ML, Servicebio). Cells were seeded in 96-well plates, and 10 μL of CCK-8 solution was added to each well, followed by incubation at 37 °C for 2 hours. The absorbance was measured at 450 nm using a microplate reader.

Western blotting

The procedure for Western blotting followed the methods described in the main text. In the supplementary experiments, the following primary antibody was additionally used: anti-cleaved caspase 3 (mouse, 1:5000, Proteintech, 68773-1).

2. Supplementary Figures

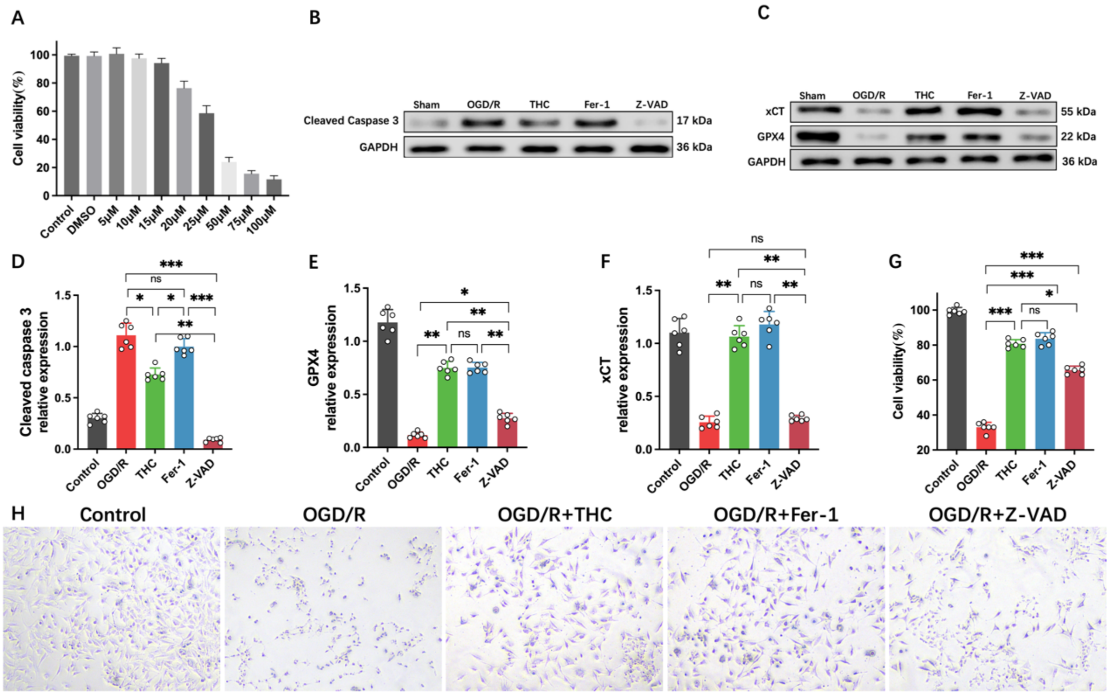

Figure S1. THC alleviates OGD/R-induced injury in bEnd.3 cells mainly through ferroptosis inhibition. (A) Cells were treated with different concentrations of THC (5–100 μM) for 24 hours,

followed by CCK-8 assay. (B-F) Western blot images and corresponding quantitative analysis of GPX4、xCT and cleaved caspase3 protein expression. (G) Cell viability of bEnd.3 cells in each group. (H) Cell morphology was observed under a microscope. All data are presented as mean ± SD, n=6 per group, one-way ANOVA, $^{*}P<0.05$, $^{**}P<0.01$, $^{***}P<0.001$.
